# Improving understanding of social prescribing by potential referrers

**DOI:** 10.3389/fpubh.2026.1760794

**Published:** 2026-04-20

**Authors:** Jason Su, Tabitha Ward, Charlotte Rafik, Matthew Cooper, Anna Purna Basu

**Affiliations:** 1 School of Pharmacy, Newcastle University, Newcastle upon Tyne, United Kingdom; 2 School of Psychology, Newcastle University, Newcastle upon Tyne, United Kingdom; 3 Population Health Sciences Institute, Newcastle University, Newcastle upon Tyne, United Kingdom; 4 Newcastle upon Tyne Hospitals NHS Foundation Trust, Newcastle upon Tyne, United Kingdom

**Keywords:** education, link worker, social determinants of health, social prescribing, terminology

## Abstract

**Background:**

Social prescribing (SP) connects people with non-clinical, community resources to address social determinants of health and improve wellbeing. Many healthcare professionals lack clear understanding of SP.

**Objective:**

To assess baseline understanding of SP among healthcare students and the effect of a brief educational video on definitional accuracy and confidence.

**Methods:**

A cross-sectional survey of Medicine, Pharmacy and Nursing undergraduates, in North-East England, assessed knowledge of SP before and immediately after exposure to a brief educational video. Participants were asked if they knew what a link worker was; their confidence in explaining SP and for a brief definition. Paired non-parametric tests assessed change.

**Results:**

49 of 76 respondents met eligibility criteria. Prior to the video 78% had heard of SP and 59% said they knew what it was but only 4 knew what a link worker was. After the video there was a significant large increase in confidence and a significant modest increase in ability to define SP. The mean definition score rose from 1.33/4 (s.d. 0.9) to 2.0 (s.d. 0.91). Two gaps in conceptual understanding persisted post-video: the referral component and the link worker role.

**Conclusion:**

Following the educational video, there were immediate improvements in confidence and modest improvements in definitional accuracy for SP. Longer term retention and application of knowledge were not assessed. Students most readily grasp “what SP connects to” (community assets, wellbeing) yet struggle with “who enacts SP and how referrals flow”. We acknowledge the small sample size. Curricula should introduce SP early and explicitly teach all components.

## 1 Introduction

Social prescribing (SP) connects people to activities, groups, and services in their community to meet the practical, social, and emotional needs that affect their health and wellbeing, usually through the support of a link worker (LW) (1). Integrating SPLWs into primary care networks allows patients across England to access SP through their general practitioner (GP) and other community services. National Health Service General Practice Workforce data for June 2025 showed that the full time equivalent of 3,390 SPLW were stationed across England (2).

The NHS England goal of referring at least 900,000 people to SP by 2023/24 (3) was surpassed in 2023 alone, with an estimated 1.3 million people referred to SP services by their GP (4). Improved outcomes from SP have been demonstrated particularly in individuals with long term conditions and mental health needs, but also in terms of the overall patient experience (5). There is evidence of positive economic impact as well as a reduction in strain on NHS services through reduced primary care and emergency department visits for frequent attenders (6).

A wide range of routes into SP exists, including referral through health and social care, and voluntary sector organizations, as well as self-referral. Evidently, optimal use of the SP system relies on awareness of its existence and knowledge of its remit. However, there are currently many challenges surrounding the referral process, including limited public awareness of the term SP (7), and knowledge gaps as well as misconceptions within healthcare services (8). These may be partly due to confusion around the varied and counter-intuitive terminology. One recent study identified 373 overlapping terms used to describe components of SP (9). This has led to some misconceptions within the medical student population as evidenced in a previous report in which only 11% of students said they had heard of social prescribing. When the concept was explained to them, many students said they had probably heard about it on their placements but not come across its “formal definition” (10). Furthermore, the overlapping terminology could lead to confusion regarding who SP benefits, what it entails and how it operates within frameworks. The term “social prescribing link worker” could mistakenly be understood as “social worker”; and the reference to “prescribing” is not automatically distinguishable from a medical prescription. These aspects of the terminology detract from the true nature and purpose of SP: indeed, a lack of shared understanding of the SP role has been identified as a barrier to its implementation (8).

Recently a consensus definition has been developed for SP, which could improve understanding of the role in the future (1). The components of this consensus definition are: referral process; connection to non-clinical community services; focus on emotional/ physical/ practical wellbeing; and implied involvement of a trusted individual within a clinical/ community setting (1). This understanding would be enhanced through incorporation of education and training on social prescribing (11), situated within a biopsychosocial healthcare lens (12). It would make intuitive sense to incorporate this training robustly but efficiently into the curricula for students within health and social care.

A brief educational video could be an effective way to introduce the concept of social prescribing. Videos have been shown to improve short- and long-term knowledge (13–15). The use of short videos in teaching has also demonstrated improved academic outcomes (16, 17). Microlearning has been defined as “an instructional approach that delivers targeted, action-oriented, bite-sized content to achieve specific objectives within a short period, typically within a few seconds or minutes”. This approach has been shown to have a positive impact on learning outcomes (18) and lends itself well to accessible, multimodal, digital delivery approaches (19). Furthermore, concise videos have been shown to increase interest and engagement in the subject (20, 21). Therefore, brief educational videos could be an effective way to merge new concepts into the curriculum of university students.

The literature already indicates that there is a lack of understanding of social prescribing amongst healthcare professionals (22, 23). Our personal experience suggests that there is also a lack of understanding of social prescribing amongst students of healthcare professions. We aimed to test this hypothesis and to demonstrate proof of concept that a brief educational video can improve conceptual understanding of social prescribing in the short term.

## 2 Methods

### 2.1 Design

A within-subjects pre - /post - exposure design was used, assessing knowledge of SP through a brief survey undertaken before and after exposure to a brief educational video.

### 2.2 Survey

Survey design and development was undertaken by JS, a Masters of pharmacy student, supervised by MC and AB. No validated survey existed which could address the specific questions posed. A range of publicly available videos on SP were reviewed for consideration for inclusion as the brief educational intervention in this study. The final video was produced by Transformation Partners in Health and Care and can be found at https://www.youtube.com/watch?v=O9azfXNcqD8. It was chosen as it succinctly summarized essential information about SP in an engaging manner. In just over 2 minutes it explains what social prescribing entails, how to access social prescribing (referral process) and the role of a link worker.

Following survey development, piloting was undertaken by JS, both supervisors and three student peers. This helped to identify and correct any errors prior to release, and to conservatively estimate completion time, which was under 10 min. The final survey structure is summarized in the Supplementary material. SurveyMonkey™ software was used for hosting of the 20-question online survey and data collection. The survey started with an informed consent process followed by demographic data collection including age, sex, degree course and year. Subsequent items covered awareness of SP and confidence in explaining it, followed by a request to provide a one-sentence definition of SP. Participants were then invited to watch the brief video explaining SP. Following review of the video, survey items revisited participant awareness and confidence in explaining SP and a repeat opportunity to provide a one-sentence definition.

### 2.3 Ethical approval and informed consent

Before study commencement, approval was obtained from the Newcastle University ethics committee (reference: 50995/2023). The study was considered low risk. Digital informed consent prior to participation was built into the survey design through an electronic participant information sheet and a consent “tick box” to proceed to the survey items.

After providing their consent participant IP addresses were collected and they were assigned a number by which they would be known for the data analysis to retain their anonymity. Furthermore, all demographic data collected was stored on a password protected computer for confidentiality and then erased at the end of the data analysis period.

### 2.4 Participants and setting

The study was conducted between 23rd October and 17th November 2024; and represents a convenience sample from a subset of healthcare students in the northeast of England, broadly representative of UK healthcare students in terms of age and sex distribution. Sample size was pragmatic, dictated by time and resource constraints, but we aimed for 50 participants. We acknowledge that this sample size limits the inferences that can be made from the results beyond demonstrating proof of concept and feasibility of the approach. Eligible participants were undergraduate students at Newcastle or Northumbria University undertaking pharmacy, medical and nursing degrees. Participants were able to access the survey through an email with a shared online link. They were asked as part of this email to send the survey to friends and peers who met the criteria, using a snowball sampling technique (24). The survey was not otherwise advertised, and we acknowledge the risk of selection bias with a snowball sampling technique. An email reminder was sent out 1 week after the original survey release. IP addresses were collected to minimize the risk of collecting multiple entries from the same participant, and the demographic data collected were reviewed as a second method of checking that each entry was from a different respondent.

### 2.5 Data analysis

Only participants with complete datasets were included in the analysis. Definitions of SP were assigned a score of 0–4 based on a rubric derived from the Common Understanding of Social Prescribing (CUSP) conceptual framework (1). The points on the rubric were: referral process; connection to non-clinical community services; focus on emotional/ physical/ practical wellbeing; and implied involvement of a trusted individual within a clinical/ community setting. Use of the word prescriber or prescribing were not considered as adequate to score a point without elaboration or explanation. Initial scoring was undertaken by JS, and then checked independently by CR and TW, with any areas of disagreement (which were minimal) highlighted and resolved by AB, though inter-rater agreement was not formally assessed. Wilcoxon signed rank tests were undertaken in SPSS [IBM statistics, version 31.0.0.0 (117)] to compare pre and post intervention scores given the nonlinear nature of the scales used.

## 3 Results

### 3.1 Participant flow and demographics

**Figure 1:**
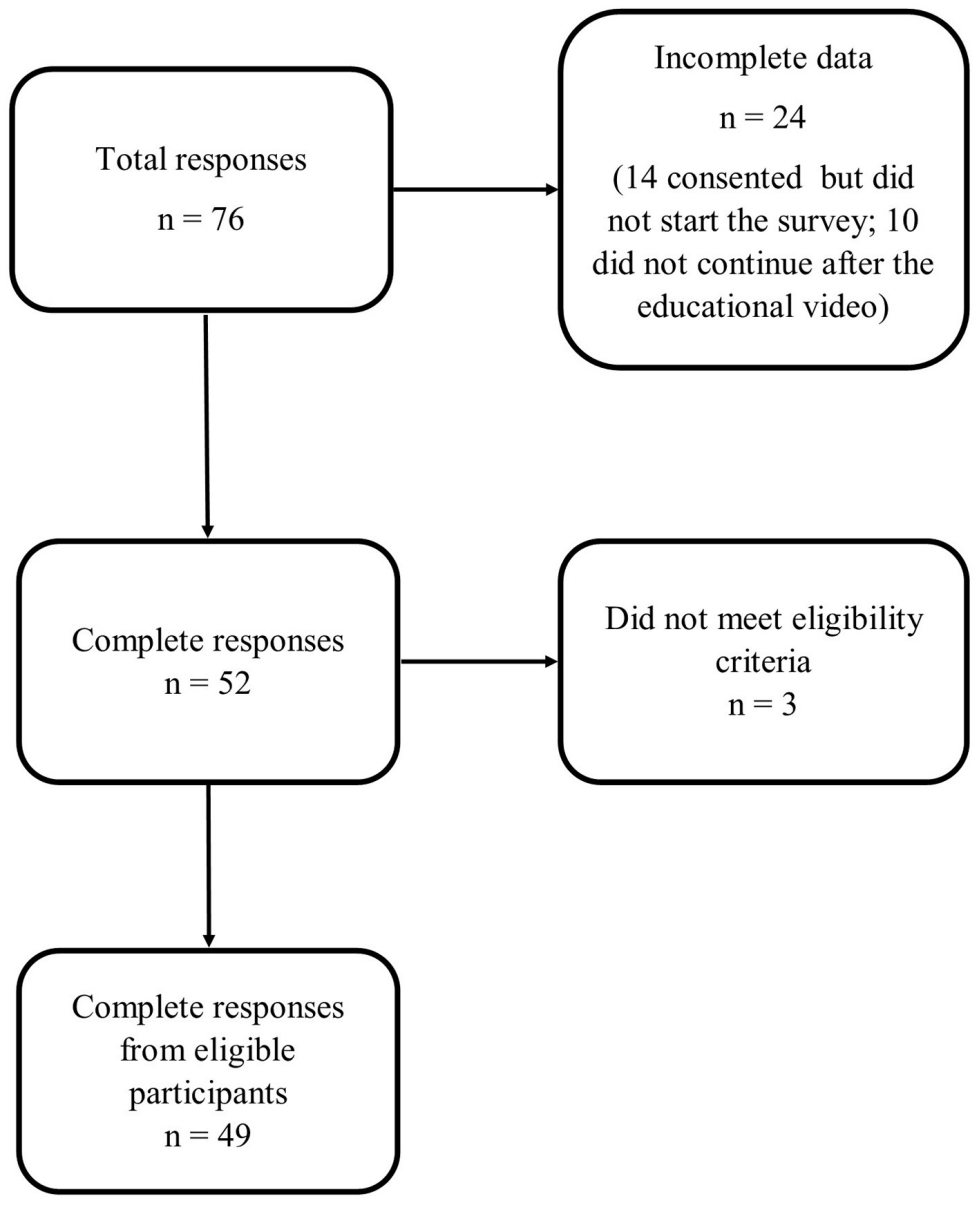
Participant flow.

Figure 1 summarizes participant flow. As a snowballing technique was used for survey distribution, the response rate could not be determined; nor do we have data on the number of participants who viewed but did not enter the survey. Of 76 respondents, 49 met eligibility criteria and provided complete responses.

The 49 participants (22 male, 27 female) were recruited from medicine (*n* = 22), pharmacy (*n* = 21), and nursing (*n* = 6). The age of participants ranged from 18 to 29 years old, with a mean age of 22 (*s.d*. = 2.05).

Twenty-nine students were in the 4th and 5th year of their degrees (4th year being the final year for pharmacy students and 5th year for medical students). All 6 nursing students were in their 2nd year.

The median time taken by all participants to complete the survey was 5 min and 25 s, though recorded completion times ranged from 2 min 21 s to 1 h 46 min.

### 3.2 Knowledge of SP

Whilst 78% (38) of the participants reported at the start of the survey that they had heard of SP, only 59% (29) said they knew what it was. Two of the participants had previously been referred to SP services by a healthcare professional. After watching the video all respondents said they understood the concept of social prescribing. Only four people stated that they knew what a link worker was, prior to watching the video. This rose to 44/49 post-video.

The mean score for the definition of SP pre-video was 1.33 (s.d. 0.9), with no difference between students in years 1–3 and years 4–5 (*z* = 0.435, *p* = 0.664, *r* = 0.0621). This rose modestly to 2.0 (s.d. 0.91) after viewing the video (*z* = 3.14, *p* = 0.002, *r* = 0.448). Table 1 shows the breakdown of scores of the definition of SP before and after the video.

**Table 1:** Analysis of participants' definitions of social prescribing.

Key component of definition	Number of individuals who included each key point	
	Before educational video	After educational video
Referral process	10	20
Connection to non-clinical community services	30	38
Focus on emotional/physical/practical wellbeing	21	36
Implied involvement of a trusted individual within a clinical/community setting	4	4

The greatest improvement (71.43%) was seen in inclusion (by 15 additional respondents) of ‘focus on emotional/ physical/ practical wellbeing' in the definitions. No change was seen in inclusion of ‘implied involvement of a trusted individual within a clinical/ community setting', which had the lowest score overall (included by only 4 respondents).

The degree of potential misconception of SP pre-video is illustrated by these explanations: “Prescribing drugs to oneself because others are taking the same drug” and “Citizens who are not medical professionals telling people to take certain drugs and not take others”. An example of a good definition post-video was: “Non-pharmacological support such as access to support groups, activity clubs or volunteers that can help you out. Access to these is facilitated through a link worker.”

The baseline mean confidence of participants in their ability to explain SP was 3.5/10: this rose to 7.1/10 after watching the video (*z* = 5.99; *p* < 0.001; *r* = 0.855).

Table 2 shows the potential domains of support through SP which were presented in multiple choice question format, broken down by the number of individuals selecting these domains before and after the educational video. The mean score pre-video was 3.18 (s.d. 0.99), increasing to 3.59 (s.d. 0.81) after the video (*z* = 3.06, *p* = 0.002, *r* = 0.438).

**Table 2:** Participant selection of potential domains of support through SP from a multiple-choice question.

Domain of support	Number of individuals who included each option	
	Before educational material	After educational material
Connecting people to local social groups	47	48
Support with housing, employment and finance	27	38
Referrals to access physical groups	43	46
Referrals to access mental health services	39	44
None of the above	2	0

Forty-seven participants (95.9%) said they found the video helpful in explaining the terms “social prescribing” and “link worker”. Only 2 disagreed, one whom left a comment asking, “Could [you] explain what a link worker does I am assuming it is the same as a social worker”. Two other people also commented that they still didn't know what a LW was.

## 4 Discussion

### 4.1 Interpretation of findings

This study aimed to assess and increase knowledge of SP in those studying to be healthcare professionals, specifically doctors, pharmacists, and nurses. Knowledge and understanding of SP, prior to exposure to a short educational video, was incomplete.

Following the educational video there was significant, large increase in participant confidence scores in being able to explain social prescribing. Furthermore, participant definitional comprehension of SP also modestly improved. The discrepancy between the two findings could perhaps be explained by social desirability effects. Specifically, confidence scores post-video could have been inflated by participants assuming that researchers expected improvements after the intervention. We do not have corroborative data to help us unpick the various possible reasons for the difference between confidence gains and conceptual mastery observed.

After viewing the video, the biggest change was an improvement in explanation of the focus of SP on improving wellbeing. Moreover, the concept with the highest number of inclusions – both pre and post video – was the non-clinical, community aspect of social prescribing. This is in line with previous findings that fully qualified healthcare professionals when asked for descriptive responses of SP, consistently mentioned “non-medical” support and wider wellbeing outcomes (25). Conversely, even after the video, most participants did not incorporate the concept of a referral process and a link worker in their explanations of SP, despite these components being described in the video.

### 4.2 Implications

The results of this study indicate that watching a short educational video about SP has the potential to improve conceptual understanding of SP in the short term. This is encouraging and suggests that addressing lack of understanding of SP within the student healthcare population is achievable despite the challenges of crowded curricula. Short videos are a valuable tool as they present essential information in a versatile and engaging way, giving them the potential to enhance course curricula and training programmes with minimal time burden. Findings from this study also add to the existing body of literature on how brief educational video interventions can improve short term knowledge (13–15) specifically definitional accuracy (13). Watching a short video has also been shown to improve long term knowledge (15), though our study did not assess this.

However, for a short video to be the most effective it must be suitable - covering all relevant content. The video we used explained that a link worker (or support broker or community navigator) as being there to “listen to you and put you in touch with whatever it is you might need in order to feel better”; and followed this up with some specific examples. The referral process to a link worker via a healthcare professional or local authority was implied but could have been stated more directly. It is surprising that it was still not clear to all students what a link worker was, after viewing the video. Overall, the video content provided a reasonable explanation of SP but clearly there are many ways that SP could be introduced and explained.

For knowledge of social prescribing to lead to patient benefit, potential referrers need to have awareness of local SP schemes and referral pathways. They also need to understand and value the importance of addressing social determinants of health where possible. The development of citizenship modules within undergraduate curricula for future healthcare professionals may assist with this, contextualizing approaches such as social prescribing within the wider educational landscape. Further exploration of the effects of the introduction of these modules should be undertaken in line with scholarship of teaching and learning methodological approaches.

Our findings suggest there has been an increase in awareness of SP over time given that a study in 2017 found that only 11% of medical students surveyed had heard of SP (10) compared with 78% of participants in our study. However, differences in setting and exact methodology preclude any direct comparison. Given the increase in use of SP schemes in the UK over this time, this is an expected trend.

### 4.3 Strengths and limitations

This preliminary study is limited by the small sample size, though this was adequate to show proof of concept of the value of a brief educational video intervention on knowledge of SP. However, the sample also has a narrow geographical spread (Newcastle/Northumbria) and did not include all groups of healthcare students (e.g., physiotherapy and occupational therapy). Therefore, we cannot be sure of the degree of generalizability of the baseline findings to students from other healthcare disciplines or from the same healthcare discipline studying elsewhere. Clearly the specific teaching program content within any one course or at any individual institution should have a bearing on what is understood about SP by students within that course or institution. Other factors such as the degree to which SP programs are advertised and available within the community are also likely to vary across the country and could influence any one student's awareness of such programs. The survey is timely nonetheless, coinciding with the planned introduction of a citizenship strand to medical undergraduate education at the hosting university, and could inform key areas necessary for reinforcement of understanding.

The survey was designed for this specific study and was not validated, which is a limitation but there was no available validated survey which could have been used instead. As the survey was undertaken online, it is possible that participants could have looked up a definition of SP to enhance their scores – however the overall quality of the definitions provided, and the short survey completion times (with a few exceptions) suggest that this was not a majority behavior.

### 4.4 Future research

Our findings suggest that improving conceptual knowledge of social prescribing is an area for inclusion in curriculum design for healthcare professional training courses in Newcastle/Northumbria. We cannot currently be sure to what extent similar issues may be occurring at other institutions. Building upon the findings of this study several key areas need to be addressed. Firstly, the role, content, and effectiveness of SP education within student curricula should be reviewed. This requires the development and use of teaching materials aligned to the specific educational aim and outcomes and to the assessment process. Consideration is required as to what are the key learning outcomes desired around social prescribing, and whether the materials address these effectively. We do not have any information about the specific teaching and learning intentions of the creators of the video we used. Based on our findings we suspect that a new brief video, designed to focus on the key points of the consensus definition of SP, would be a valuable addition to the microlearning armamentarium for healthcare students (1). This could then be aligned to assessments based on that definition, and longitudinal and or/controlled studies undertaken to explore effectiveness. Other aspects of learning around social prescribing are likely to require different pedagogical approaches.

The short-term improvement seen with a brief video suggests that even with the pressures on curriculum content, some aspects of education on SP can be incorporated through microlearning (18, 19). Clearly there also needs to be enough exposure to achieve longer term knowledge retention. In addition, knowledge transfer ideally needs to be supplemented by practical exposure to SP, communication skills development, and an enlightened attitude toward supporting the holistic needs of patients, with awareness of the importance of addressing social determinants of health where possible.

## 5 Conclusion

We have demonstrated the feasibility of using a microlearning approach to improve conceptual understanding of social prescribing by healthcare students. Baseline conceptual understanding of SP in the students assessed was incomplete. Following the brief educational video intervention, there were immediate improvements in confidence and modest improvements in definitional accuracy for SP, representing partial conceptual gains. Longer term retention and application of knowledge acquired were not assessed within this study. Students most readily grasp “what SP connects to” (community assets, wellbeing) yet struggle with “who enacts SP and how referrals flow”. We acknowledge the small sample size as a study limitation. Curricula should introduce SP early and explicitly teach all components using pedagogical methods tailored to the specific aims, reviewing the effectiveness of approaches used. Our hope is that improved understanding of SP by healthcare students would form a solid basis for optimal referral practices to SP services in their professional careers.

## Data Availability

The raw data supporting the conclusions of this article will be made available by the authors, without undue reservation.
