# Supplementary material for: Improving understanding of social prescribing by potential referrers
Source: Front Public Health. 2026 Apr 20;14:1760794. doi: 10.3389/fpubh.2026.1760794 (PMC13136140; doi:10.3389/fpubh.2026.1760794)
Supplement: Supplementary file 1 [file Table_1.docx]

**Supplementary Material: Survey structure**

| Section of the survey | Question number | Question |
| --- | --- | --- |
| Consent | 1 | By pressing "Next" you are giving your consent to participate in this study. If you are happy to continue, please confirm by ticking the box “Next” |
| Demographics | 2 | How old are you? |
|  | 3 | What is your sex? |
|  | 4 | What are you studying? |
|  | 5 | What year are you currently in? |
| Pre- video | 6 | Have you heard the term ‘Social Prescribing’ before this survey? |
|  | 7 | Do you know what social prescribing is? |
|  | 8 | If yes, please explain in one sentence what social prescribing is. |
|  | 9 | If no, based in the term ‘social prescribing’ what do you think it involves? |
|  | 10 | How confident did you feel in explaining social prescribing? (1-10) |
|  | 11 | Have you ever been referred for social prescribing by a healthcare professional? (yes/no) |
|  | 12 | Do you know what a link worker is? |
|  | 13 | Which of these options do you think social prescribing could include:   - Connecting people to local support groups - Support for housing, employment and finance - Referrals to access physical groups - Referrals to access mental health services - None of the above |
| Educational video | | |
| Post video | 14 | Do you understand the concept of social prescribing? |
|  | 15 | Could you have another go at explaining what social prescribing is in one sentence? |
|  | 16 | How confident did you feel when you were explaining 'social prescribing'? (1-10) |
|  | 17 | Do you know what a link worker is? |
|  | 18 | Which of these options do you think social prescribing could include:   - Connecting people to local support groups - Support for housing, employment and finance - Referrals to access physical groups - Referrals to access mental health services - None of the above |
|  | 19 | Was the video helpful with explaining the terms 'social prescribing' and 'link worker'? |
|  | 20 | Do you have any other comments? |
